# Experimental Observation of Isolative Efficacy of a Solid Coupling Medium in Extracorporeal Shock Wave Lithotripsy—Implications to Nosocomial Infection Prevention

**DOI:** 10.3390/pathogens11101103

**Published:** 2022-09-27

**Authors:** Hui-Wen Chou, Chih-Lin Huang, Yu-Chih Lin, Yusen Eason Lin, Wei-Chuan Chen

**Affiliations:** 1 Department of Laboratory Medicine, Kaohsiung Medical University Hospital, Kaohsiung 807377, Taiwan; 2 Division of General Internal Medicine, Department of Internal Medicine, Kaohsiung Medical University Hospital, Kaohsiung 807377, Taiwan; 3 Department of Medical Humanities and Education, Kaohsiung Medical University, Kaohsiung 807377, Taiwan; 4 Graduate Institute of Human Resource and Knowledge Management, National Kaohsiung Normal University, Kaohsiung 802561, Taiwan; 5 CleanWave Medical Co., Ltd., Kaohsiung 806613, Taiwan; 6 Department of Medical Education and Research, Kaohsiung Veterans General Hospital, Kaohsiung 813414, Taiwan; 7 Division of Urology, Department of Surgery, Kaohsiung Veterans General Hospital, Kaohsiung 813414, Taiwan

**Keywords:** isolation, coupling, extracorporeal shock wave lithotripter

## Abstract

**Introduction:** Extracorporeal shock wave lithotripsy (ESWL) is a well-established, popular treatment choice for renal stones. Traditionally, the semi-liquid gel is used as a coupling medium in ESWL. During ESWL, body fluid or blood might transmit between the patients when the probe or gel used in the procedure is contaminated and cause potential nosocomial infections. To solve this problem, we developed a solid coupling medium (isolation coupling pad, referred to as “icPad”) between the patient’s skin and the probe as a shock wave transmission medium to prevent contamination. This study aimed to investigate the isolative efficacy of the icPad in blocking the permeation of microbes. **Method:** Rhodamine 6G (a fluorescent dye) was used as a tracer to simulate the microorganisms. The penetration of the fluorescent dye on the longitudinal section of the icPad was observed by a microscope after the dye was placed on the body side of the icPad for 40 min. After the shock wave, icPad was extracted with 75% ethanol, and fluorescence intensity was measured with a fluorescence spectrometer. **Results:** Our results revealed that the body side of icPad is free of fluorescent dye during lithotripsy. Qualitative analysis results confirmed that icPad has an isolative effect on simulating contaminants such as bacteria or viruses. **Conclusion:** In this in vitro phantom study, a proprietary icPad can be an isolative coupling medium and is speculated to avoid cross-contamination of bacterial or viral infection during ESWL.

## 1. Introduction

Urinary tract stones are a common clinical disease. Approximately 12% of the global population suffers symptoms from urinary tract stones [1]. The prevalence of urinary tract stones in Taiwan is 9.6% [2]. Extracorporeal shock wave lithotripsy (ESWL) is the preferred method of treating urinary tract stones [3]. In ESWL, semi-liquid gel (referred to as “gel”) is used as a coupling medium between the skin and the lithotripsy probe so the shock wave can transmit into the target area [4]. As for the success rate of ESWL in treating urinary tract stones, the coupling interface is an important factor [5,6,7].

However, when the physician performs the ESWL procedure, there may be an outflow of body fluids and bloody mixture in the gel and on the probe surface if the probe contracts a wound on the patient’s skin. Without a thorough cleaning standard, it can cause cross-contamination between patients through contact with body fluids and blood. The literature has reported that the gel used in ultrasound procedures is a potential source of infection that causes nosocomial outbreaks [8], and ultrasound probes may serve as a medium for infection transmission [9]. This finding implies that cross-contamination between patients may also occur during EWSL procedures; however, the conventional disinfection methods (e.g., alcohol, solvent, aldehyde, or oil) are not effective against contamination on the lithotripsy probe. According to the lithotripsy manufacturer (Dornier MedTech Europe GmbH Co., Weßling, Germany), the surface of the probe is an essential component, and may be damaged by improper disinfectant. Although some studies have suggested that using a non-sterile dry tissue to wipe the ultrasonic probe can reduce bacterial contamination by more than 50% [9], it still cannot achieve a contamination-free and disinfected condition to prevent nosocomial infections. Thus, finding a method that can effectively prevent any fluid from the patient’s body from the lithotripsy probe is an ongoing challenge.

A newly designed isolation coupling pad (referred to as “icPad”) was recently introduced for ESWL as an alternative to gel [10]. icPad is a solid shock wave transmission medium between the lithotripsy probe and the human body. The pad has efficient shock wave transmission and adhesive properties by reducing air pockets in the coupling area. icPad has documented efficacy to demonstrate better shock wave transmission, a lower number of shock waves required, and an increased rate of stone fragmentation [11]. 

Rhodamine 6G is a fluorescent dye with good photostability and quantum yield. It can be used in the field for water flow tracking, leak testing, fluorescence microscopy, or dye lasers [12]. Thus, we want to use it to test the barrier efficacy of icPad against microorganisms. Rhodamine 6G has a molecular weight of 479.01 g/mol and a polar surface area (PSA) of 0.615 nm^2^ [13], which is much smaller than viruses and bacteria [14]. Therefore, it is reasonable to infer that if icPad can block the penetration of Rhodamine 6G, it could also block the penetration of bacteria and viruses to reduce the risk of cross-contamination. 

The purpose of this study is to determine the efficacy of the icPad, which could isolate the direct contact between the probe and the patient’s body and then block the permeation of contaminants during the ESWL procedure. The finding in this study may indicate its clinical application to cross-contamination and potential infections among patients during ESWL.

## 2. Materials and Method

### 2.1. Test Material

**Figure 1:**
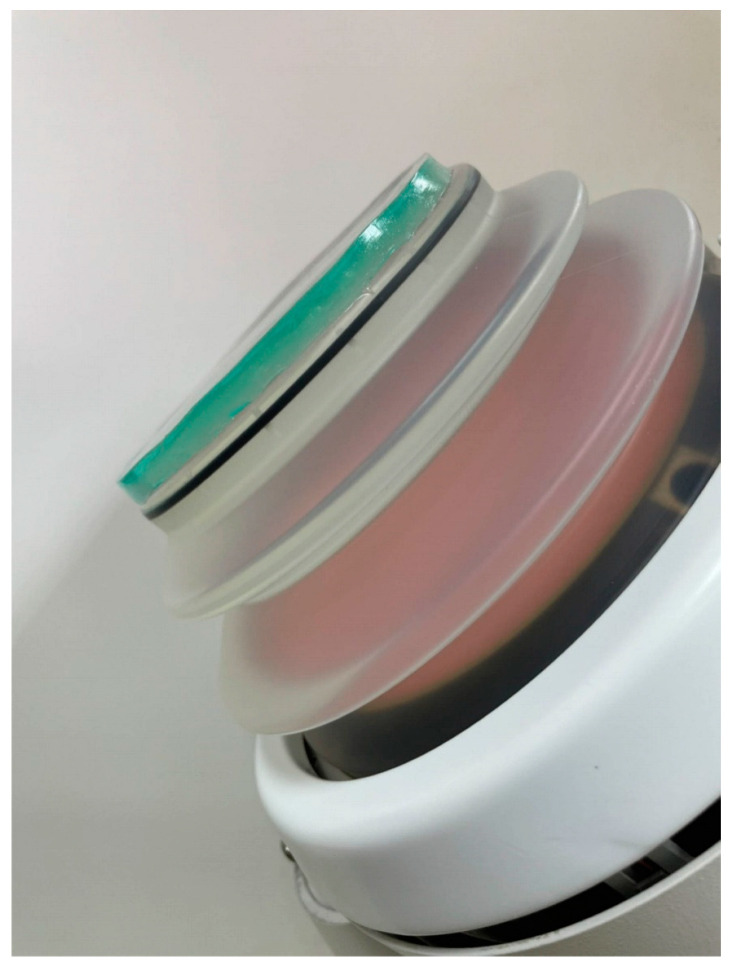
icPad sticking on the treatment head.

Coupling Medium: The main component of the isolation coupling pad (icPad) is polyacrylamide with a diameter of 120 mm and a thickness of 8 mm [11]. There are two sides of icPad, the A-side and B-side; the A-side is attached to the EWSL probe, and the B-side is attached to the patient body (Figure 1). 

Contaminant-Simulating Substance: Rhodamine 6G was purchased from Tokyo Chemical Industry Company (Japan). Ethanol was bought from Sigma-Aldrich company (USA).

Equipment: Spectrofluorometer FS5 (Edinburgh Instruments, Livingston, United Kingdom). 

### 2.2. Experimental Procedure

The dye penetration through the icPad is determined by two quantitative observations: microscopy and spectrofluorometer. The concentration of 1 mg/mL of the Rhodamine 6G solution (“dye”) is used throughout the study. The presence of dye is measured by a spectrofluorometer at excitation wavelength 525 nm and emission wavelength 549 nm.

**Figure 2:**
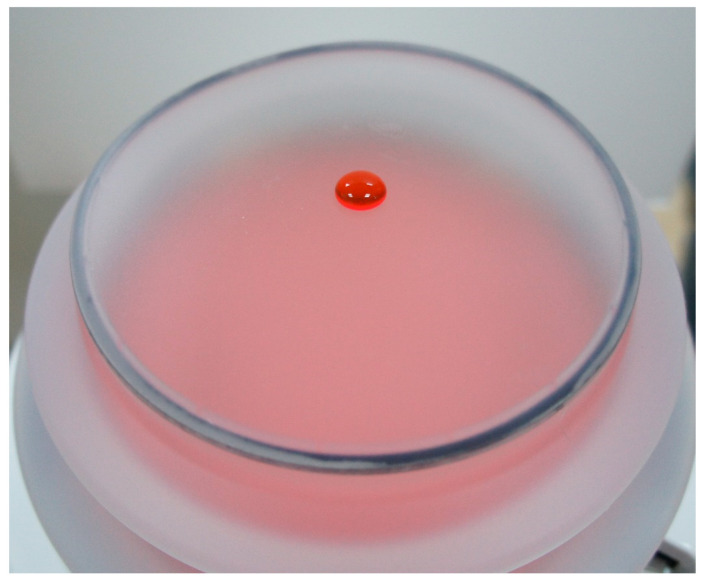
A drop of fluorescent dye simulating blood and virus contaminant on the probe surface of lithotripter.

**Figure 3:**
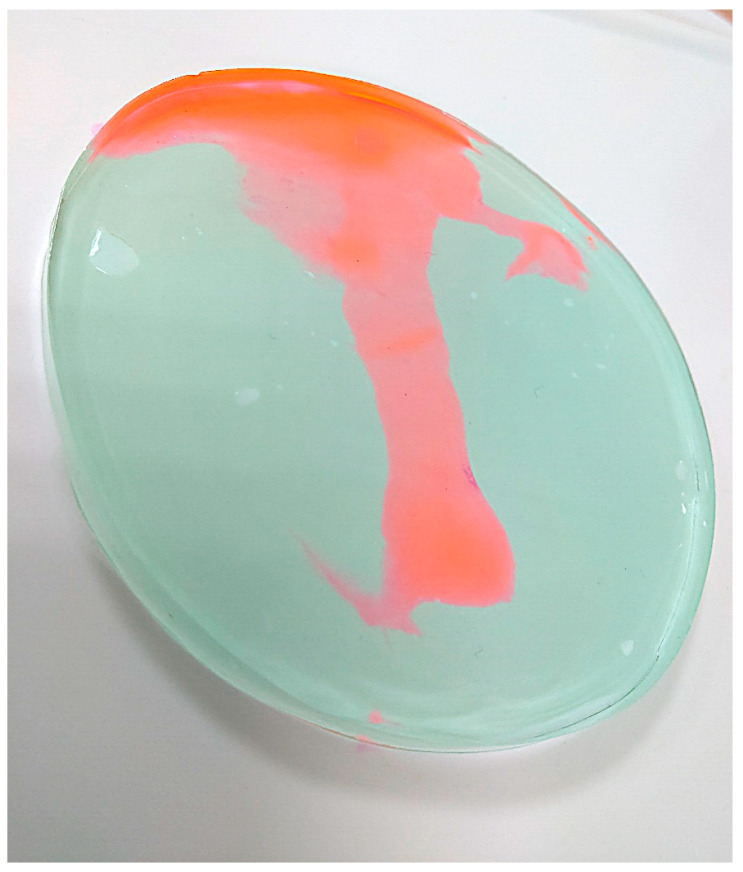
Fluorescent dye was diffusively distributed on the probe surface.

Microscopy Observation: To determine how far the dye would penetrate the icPad, 0.3 mL of dye was placed onto the probe’s B-side of the icPad attached to the probe (Figure 2). Then the fluorescent dye was diffusively distributed on the A-side of another icPad (Figure 3). After 40 min of contact, the section of the B-side with dye was cut into 1 mm square slices longitudinally and placed under a 20× and 40× microscope (Olympus CX43). The penetration distance of dye through the icPad was measured in millimeters.

**Figure 4:**
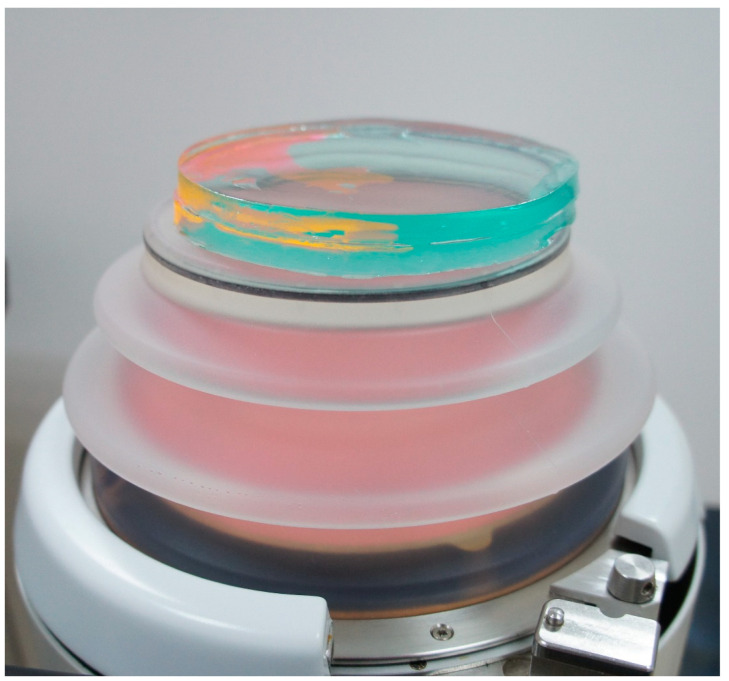
icPad simulating body skin was pasted on to the body side of interfaced icPad.

**Figure 5:**
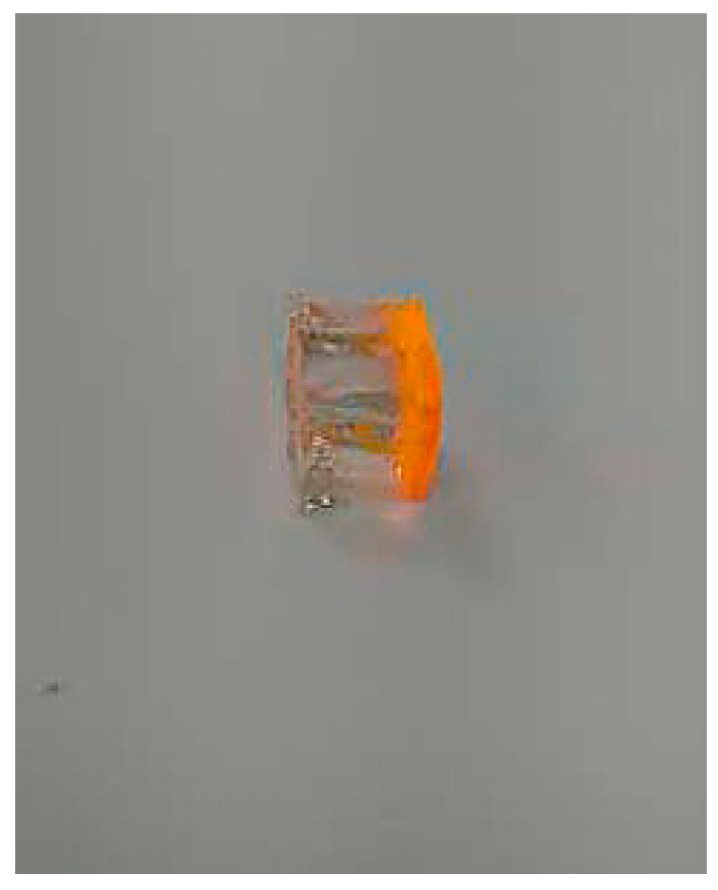
icPad of length 10 × width 10 × depth 4 mm^3^ was cut for extraction of fluorescence dye.

Spectrofluorometer Observation: To determine how far the dye would penetrate the icPad during an ESWL procedure, the experiment was divided into three groups (control, test, and blank groups). Next, 0.3 mL of dye was placed on the B-side of the icPad in the control and experimental groups, and the blank group remained free of dye. Then the A-side of icPad from the experimental group was attached to the probe. In the meantime, another icPad that simulated the human body was applied on the B-side of the icPad from the experimental group to absorb, if any, the possible penetrating fluorescent dye on the B-side (Figure 4). ESWL was then performed to simulate the renal stone treatment at shock wave set at 80 hit/min and energy level four. The total pulses of shock wave were 3000. After ESWL, icPad was cut into 10 × 10 × 8 mm^3^ (Figure 5) and extracted in 75% ethanol alcohol for 30 min. In the control group, the experiment was performed the same as the experimental group except without placing dye on the B-side of icPad. In the blank group, icPad without applying the fluorescent dye was placed as the control for background fluorescence intensity. The fluorescence intensity was measured by a spectrofluorometer. This experiment was conducted in triplicate.

## 3. Result

### 3.1. Microscopy Observation

**Figure 6:**
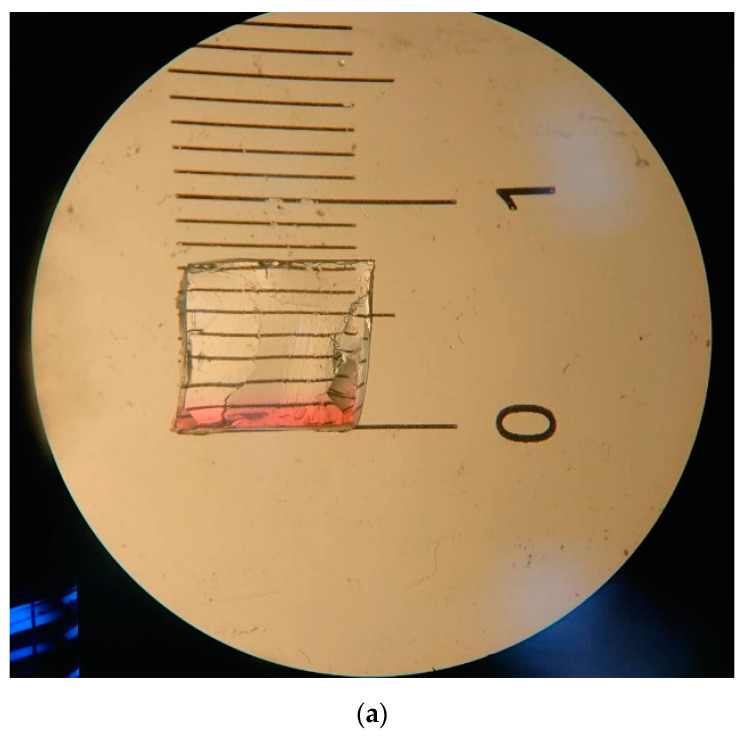

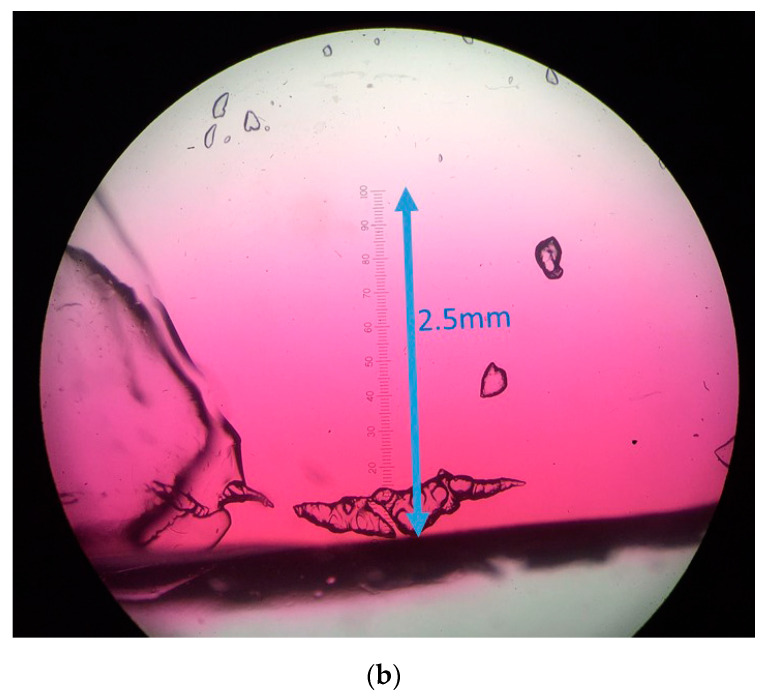
Permeation of fluorescent dye was shown on the icPad, (**a**) (20×) and (**b**) (40×).

After the fluorescent dye was attached to the icPad, it was allowed to stand at room temperature for 40 min, and then the sections were observed under a 20× and 40× microscope. The distance of fluorescent dye permeation on the surface of the icPad was 2.5 mm (Figure 6a,b), which was less than the thickness of 8 mm of the icPad. Due to the force of penetration being only 32% (2.5 mm/8 mm), the dye could hardly penetrate the icPad and come into direct contact with the human body during an ESWL procedure.

### 3.2. Spectrofluorometer Observation

We performed an ANOVA analysis of the difference in fluorescence intensity (count per second; CPS) of the experimental, control, and blank groups. The results showed no significant difference in fluorescence intensity among the three groups (F = 0.048, *p* > 0.05) (Table 1). The fluorescence intensity after the shock wave has no significant difference from blank (background intensity). This suggests that the fluorescent dye did not penetrate the icPad after ESWL. 

## 4. Discussion

A critical treatment method for urinary calculi, interface coupling, is a factor in therapeutic outcomes [7]. Air pockets covering 1.5~19% of the coupling area may reduce the shock wave amplitude by 20% [15]. Air pockets would decrease the therapeutic effect of ESWL [16]. The icPad has been documented experimentally with the advantages of reducing the number of the shock wave and increasing stone disintegration rate because of reducing trapped air pockets in ESWL coupling [10,11]. However, the capability of icPad in preventing the cross-contamination of skin infection is unknown despite its superior coupling efficacy. Compared to ultrasound, the contact infection of the lithotripter probe is similar or even worse if the skin barrier is disrupted by the shock wave during ESWL. Because complications following ESWL, including bruising (up to 26%) and blood in the contact gel, are common [17,18,19], there is a possibility of an outflow of body fluids or bloody mixture on the gel and the probe. Given that the probe uses semi-liquid gel as an interface, the patient skin may be a potential source of nosocomial infection [8]. Therefore, it is noteworthy that this research intended to investigate the isolative efficacy of the icPad. 

The microscopy observation showed that the depth of the fluorescent dye after 40 min of permeation was only about 2.5 mm, and the high barrier of 8 mm then could speculate about blocking the contaminants. In addition, the quantitative analysis also revealed that after the shock wave for 40 min, the intensity of fluorescein dye was not significantly different among the experimental, control, and blank groups, which means that icPad could be used as a coupling and isolation pad. Since the PSA of the fluorescent dye used in this study is about 0.615 nm^2^, much less than viruses and bacteria [13,14,20], if the material of this size did not permeate the icPad, it thus indicates that the 8 mm icPad may act as a barrier to prevent penetration of contaminants. In short, both results confirmed that the surface of the body is free of contamination after the use of icPad as a coupling medium during lithotripsy, and the cross-contamination of skin infection could be avoided. 

This experimental observational study is the first investigation of the isolation efficacy of icPad during ESWL lithotripsy. However, since this is an in vitro phantom study, the biological nature of the infectious microorganisms was not applied in this study. To achieve the isolative effectiveness of icPad, an in vivo study is needed to confirm the conclusion of this study.

## Figures and Tables

**Table 1:** Fluorescence intensity between different groups.

Group.	No. of Sample	Mean *	±S.D.	*p* Value **
Experimental	3	0.219	0.083	0.953
Control	3	0.192	0.100	
Blank	3	0.198	0.147	

* Unit: Fluorescence intensity (CPS/10^6^) ** *p* > 0.05.

## Data Availability

Not applicable.
